# Sialorrhea in Parkinson’s Disease

**DOI:** 10.3390/toxins12110691

**Published:** 2020-10-31

**Authors:** Jonathan Isaacson, Sanskruti Patel, Yasar Torres-Yaghi, Fernando Pagán

**Affiliations:** Department of Neurology, Medstar Georgetown University Hospital, 2800 Reservoir Rd., NW Washington, D.C., WA 20007, USA; Jonathan.R.Isaacson@gunet.georgetown.edu (J.I.); Sanskruti.Patel@medstar.net (S.P.); Yasar.Torres-Yaghi@gunet.georgetown.edu (Y.T.-Y.)

**Keywords:** sialorrhea, botulinum toxin, Parkinson’s disease, drooling, Abobotulinumtoxin A, Incobotulinumtoxin A, Onabotulinumtoxin A, Rimabotulinumtoxin B

## Abstract

Sialorrhea, or excessive saliva beyond the margin of the lip, is a common problem in many neurological diseases. Previously, sialorrhea has been underrecognized in Parkinson’s disease (PD) patients. Despite this, many patients rank sialorrhea as one of the most debilitating complaints of Parkinson’s disease. Previous treatment for sialorrhea has been suboptimal and has been plagued by significant side effects that are bothersome and can be dangerous in patients with a concurrent neurodegenerative disease. This review sought to review the anatomy, function, and etiology of sialorrhea in PD. It then sought to examine the evidence for the different treatments of sialorrhea in PD, and further examined newer evidence for safety and efficacy in minimally invasive treatment such as botulinum toxin.

## 1. Introduction

Sialorrhea, commonly referred to as drooling, is defined as excessive saliva beyond the margin of the lip. Sialorrhea is caused by either hypersalivation or problems with the removal of saliva considered abnormal after the age of 4. It is a common neurological manifestation of many neurological diseases including cerebral palsy (CP), Parkinson’s disease (PD), and amyotrophic lateral sclerosis (ALS) [1]. Drooling can be a devastating and debilitating complication of PD and is one of the most prevalent complaints of patients. It is often underrecognized and undertreated. Some estimates show that up to 80% of patients with Parkinson’s disease experience sialorrhea [2]. In advanced PD, it is among the three most common complaints from patients overall [3]. Drooling is very problematic and can lead to sheer embarrassment, social isolation, depression, skin infection, malodor, and aspiration pneumonia [4]. Accumulation of secretions puts patients at an increased risk of aspiration of the products, and thus can lead to significant morbidity and mortality associated with aspiration pneumonia in patients with PD [5]. Aspiration pneumonia carries around a 20% mortality in PD patients [6].

## 2. Etiology of Sialorrhea in Parkinson’s Disease (PD)

Initial hypotheses suggested that sialorrhea was attributable to an excessive production of saliva secondary to autonomic dysfunction, but further sialometry studies showed that production was actually reduced in PD versus non-PD patients [7]. Thus, thought shifted to the likely etiology being a decreased ability to swallow, thereby interfering with the clearance of saliva, which is reliant upon adequate muscle coordination to initiate the swallow reflex. There are three phases to swallowing: an oral phase under voluntary control, a pharyngeal phase, and an esophageal phase, where the latter two are under involuntary control. One study by Umemoto et al. evaluated quantitative video-fluoroscopic images of PD patients to demonstrate if the oral phase worsened with the progression of PD [8]. They measured the speed of movement of barium gelatin jelly and the associated range of motion of oropharyngeal muscles. The study result showed that maximum tongue pressure was significantly larger, and oropharyngeal transit time was significantly shorter in mild to moderate (Hoehn and Yahr stages II and III) PD versus advanced (Hoehn and Yahr stages IV and V) PD. They also found a significant negative correlation between the speed of oropharyngeal muscle movement and the resulting transit time, which reinforced the hypothesis that impaired oropharyngeal transport and reduced swallowing frequency lead to oral phase deterioration [8]. Additional studies associated sialorrhea with higher UPDRS motor scores, loss of motor function, and progression of PD. Thus, the current literature suggests sialorrhea is likely caused by a combination of oropharyngeal bradykinesia and inability to clear salivary secretions, which together lead to the excessive pooling of saliva seen in PD patients [9].

## 3. Salivary Glands

The major salivary glands in the oral cavity are the parotid, submandibular, and sublingual glands. They have multiple functions, including lubrication, immunity, dental health, digestion, and overall maintenance of homeostasis. The average daily flow of saliva is typically 1 to 1.5 L a day. The parotid gland is the largest salivary gland and produces the most saliva. It is composed of serous secreting glands and is in the preauricular region along the posterior surface of the mandible. It is divided into two lobes: the superficial and the deep, which are separated by the facial nerve. The secretion of saliva is triggered by mechanical, gustatory, and olfactory stimuli [10]. The parotid gland produces salivation and its secretomotor function is controlled by the parasympathetic nervous system via the postganglionic parasympathetic fibers arising from the Otic ganglion. The preganglionic parasympathetic neurons are located in the inferior salivatory nucleus in the brain stem and reach the Otic ganglion via the glossopharyngeal nerve (CN IX) and its branches.

The second largest salivary gland is the submandibular gland, which has a mixed mucous and serous function. Parasympathetic innervation to the submandibular glands is provided by the superior salivatory nucleus via the chorda tympani, a branch of the facial nerve. The sublingual gland is the smallest salivary gland and is in the anterior floor of the mouth and secretes only mucous fluid. The nervous supply of the sublingual gland reflects that of the submandibular gland. In the unstimulated state, only 20 percent of the flow is from the parotid, whereas most of the secretions are produced by the submandibular and sublingual glands, 65% and 8%, respectively [1,11]. In the stimulated state, saliva production is increased 5× with the vast majority produced by the parotid gland. In the unstimulated state, normal flow is around 0.3 mL/min, with anything greater than 0.1 mL/min considered normal. In the stimulated state, flow ranges from 0.2 up to 7 mL/min, and is responsible for as much as 80–90% of average production [11].

## 4. Conservative Management and Speech Therapy

Noninvasive management, which includes speech-language therapists, dentists, and physiotherapists, is often the first step in management for most providers, but unfortunately, these measures have not shown significant long-term benefits. Numerous previous studies focused on interventions by speech language therapists such as cueing and prompting the patient to swallow. Although an initial benefit was noted, the degree of improvement was not maintained at 3 months [12]. Furthermore, conservative methods rely on independence and behavioral modifications with self-care of sialorrhea, which can become very difficult in patients with concurrent neurodegenerative disease. In a pooled analysis of multiple studies evaluating speech therapy for sialorrhea management, the vast majority showed a decrease in drooling, but did not achieve long term effects [13]. The behavioral and self-management therapy needed to be maintained and patients needed to be independently motivated to continue the exercises. These noninvasive techniques need significant longitudinal support and consistent effort, and thus are limited by practicality, time consumption, and fading of effect. Conservative measures can also be pursued including neck collars and head back wheelchairs, optimizing dopaminergic therapy, hydration, and ensuring oral rehabilitation [14]. A study by South et al. demonstrated that chewing gum may modify certain swallowing parameters and reduce drooling in PD patients [15]. These tend to target symptom control, but have less effect on modifying the progression of the drooling. Noninvasive management may thus be effective as an adjunct therapy along with medical intervention [16].

## 5. Oral Therapy

Oral medication therapies for sialorrhea have often been the mainstay treatment for sialorrhea, but use of these medications is often limited by significant and heterogenous side effects, black box warning in the elderly, and suboptimal benefit. Typical oral therapy includes anticholinergic drugs such as hyoscine, scopolamine, glycopyrrolate, and ipratropium. Anticholinergic drugs work by blocking cholinergic receptors, and thus decreasing the production of saliva by inhibiting cholinergic parasympathetic and postganglionic sympathetic activity. Less production of saliva reduces the burden of excretion in the setting of oropharyngeal bradykinesia. Unfortunately, these medications are not specific to only muscarinic receptors and thus can result in multiorgan adverse effects, especially in patients with neurodegenerative disorders [14].

Glycopyrrolate has been the most studied anticholinergic for sialorrhea, and is the preferred option given that it has minimal central nervous system (CNS) effects, and exerts most of its effects on salivary secretion and sweat gland activity [17]. A new formulation of glycopyrrolate (Cuvposa) has received FDA approval to treat chronic severe drooling in children ages 3 to 16. Glycopyrronium bromide is an anticholinergic drug with a quaternary ammonium structure, which limits its ability to cross the blood brain barrier. That said, the spectrum of CNS adverse effects can range from drowsiness to hallucinations, to severe cognitive impairment, to possibly coma [18]. In a study by Arbouw et al., it was found that the glycopyrrolate in PD patients had a modest improvement in mean sialorrhea score from 4.6 to 3.8, with no significant adverse side effects [19]. In other studies, the use of anticholinergics was limited because of dry mouth, skin reaction, confusion, behavioral changes, sleep disturbances, constipation, and urinary retention [13]. Anticholinergics are often poorly tolerated in PD and neurodegenerative diseases. Patient’s with PD typically have some degree of autonomic dysfunction, and thus poorly tolerate anticholinergic medications. Moreover, given the mostly elderly population in PD and the comorbid cognitive impairment related to the disease, patients are much more prone to cognitive side effects of anticholinergics [14]. In a summative review of the literature, no significant improvement in sialorrhea was reported in comparison with placebo in elderly patients [19,20].

## 6. Botulinum Toxin

In 1820, Dr. Justinus Kerner, a small-town German medical officer and romantic poet, gave the first complete description of clinical botulism based on extensive clinical observations of so-called “sausage poisoning”. He observed that the toxin develops under anaerobic conditions and can be lethal in minute doses. His prescience in suggesting that the toxin might be used therapeutically earned him recognition as the pioneer of modern botulinum toxin therapy. It was not until the 1980s that the therapeutic benefit of this toxin became apparent, and it began to have use in many medical applications. Botulinum toxin is composed of both a neurotoxin component and a non-toxic component. The neurotoxin component is made up of a light chain (50 kDa) and a heavy chain (100 kDa) attached together by a disulfide bond. The non-toxic proteins are composed of a hemagglutinin complex and non-hemagglutinating proteins. Botulinum toxin is produced by Clostridium botulinum. There are seven different serotypes of botulinum neurotoxin (A, B, C, D, E, F, G) [21]. All serotypes interfere with the neural transmission by blocking the release of acetylcholine at the neuromuscular junction at the presynaptic neuron. 

**Figure 1:**
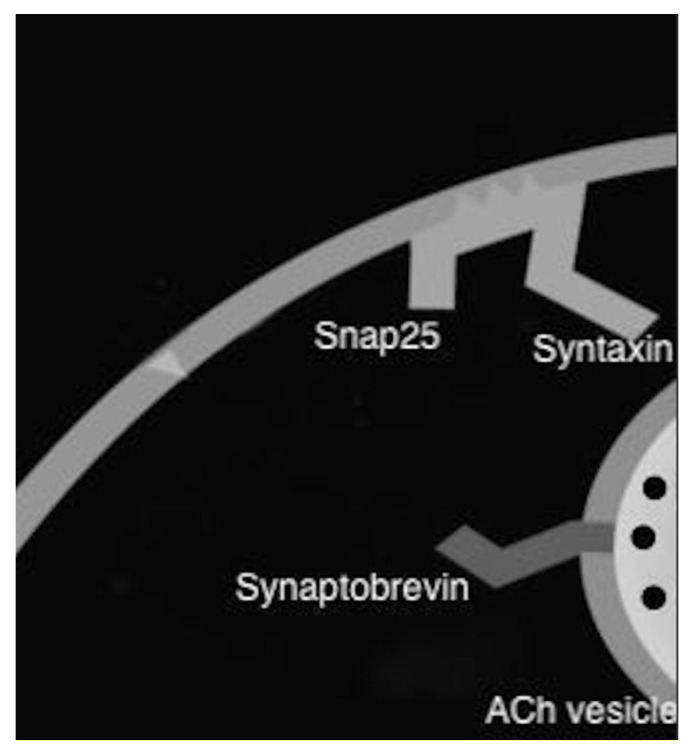
Mode of action of botulinum toxin.

When a neuron depolarizes down an axon terminal, acetylcholine is released into the synaptic cleft. The ability for acetylcholine to be released is performed by a docking protein complex called SNARE (soluble N-ethyl-maleimide-sensitive factor attachment protein receptor) that assists with vesicle fusion, and then releases it into the nerve terminal. After intramuscular injection, tissue protease nicks the toxin and detaches the toxin molecule from the protein complex within minutes. The heavy chain has selective irreversible affinity for presynaptic cholinergic neurons. The heavy chain of the toxin attaches the toxin to the outer surface of the presynaptic membrane at specific polysialogangioside receptors. The toxin is then taken up into the neuron by endocytosis, and the light chain and the heavy chain are cleaved, allowing the toxin to be released into the cytoplasm. The light chain of the neurotoxin binds with high specificity to a SNARE protein complex, and thus prevents the docking of acetylcholine into vesicles. Each serotype has a different target and mode of action. Botulinum toxin A cleaves SNAP25. Botulinum toxin B cleaves vesicle-associated membrane protein (VAMP) or Synaptobrevin II. The cleavage of these proteins prevents docking and fusion of vesicles, and prevents the release of acetylcholine into nerve terminals, causing paresis [21]. This is illustrated in Figure 1 below; the light chain of the botulinum toxin cleaves Snap 25 (botulinum toxin A) or Synaptobrevin (botulinum toxin B) and prevents docking and release of Acetylcholine. The toxin requires 48–72 h to take effect, and the peak effect is usually around 10–14 days. The effect of the toxin lasts between 8 and 12 weeks [22]. The effects diminish as SNARE proteins are restored. There is correlation between the dose of botulinum toxin and the amount of paresis, but significant paresis is seen with even low doses and there is only modest additional benefit with higher doses. There is also a correlation between the duration of effect and dose, but once an adequate dose is met, the duration tends to maximize at around 12 weeks [21]. This is likely because the long duration of effect of the toxin is less from the half-life of the toxin, but more so from the slow process of both nerve terminal regeneration and new extra-junctional synapses [23].

## 7. Botulinum Toxin in Sialorrhea

Botulinum toxin has class A evidence in the treatment of sialorrhea. Both botulinum toxin A and B have been used in the treatment of sialorrhea, and have been shown to have fewer side effects than comparative treatment [24]. By injecting botulinum toxin locally into the salivary glands, the production of saliva is reduced, and the risks of systemic effects are limited. Botulinum toxin A was first used by Pal et al., who showed a marked improvement in the reduction of saliva and a 66% subjective improvement in patients [25]. In 2004, a study by Ondo et al. showed similar efficacy of botulinum toxin B in the treatment of drooling [26]. Further pivotal studies have led to botulinum toxins becoming FDA-approved therapies for the treatment of sialorrhea, particularly serotypes IncobotulinumtoxinA (07/0218) and RimabotulinumtoxinB (08/2019).

The therapeutic uses of botulinum toxin A and B are well recognized and rapidly expanding. There are three botulinum A toxins: AbobotulinumtoxinA, IncobotulinumtoxinA, and OnabotulinumtoxinA, and one botulinum B toxin, RimabotulinumtoxinB. Each work in slightly different ways and have different indications. All four agents differ in complexity, purity, potency, dosing, and immunogenicity [27]. Key differences between the four major botulinum toxins are listed in the Table 1 below.

## 8. Techniques 

There are two main accepted ways for localization of the salivary glands for botulinum toxin treatment: anatomical or ultrasound guided. The ultrasound guided approach targets the maximum gland thickness, whereas the anatomical approach uses known landmarks and positioning based on published recommendations. For parotid gland localization, the FDA recommended approach is to locate the midpoint between the tragus and the angle of the mandible, and to deliver an injection 1 cm anterior to this point. For the submandibular gland anatomic localization, the recommendation is to find the midpoint between the angle of the mandible and the tip of the chin and to inject 1 finger breadth medial to the inferior surface of the mandible.

Both are approved approaches, although some argue that an ultrasound guided approach may allow for more accurate injection into the gland [30]. In some studies, the success rate for salivary gland injection via anatomical landmark of parotid and submandibular glands ranged from 30 to 70%. The success rate varied greatly based on population, type of study, which glands were targeted, dosage, and level of training and technique. In another study, the accuracy of anatomical versus ultrasound guided approaches into the parotid gland in cadavers was 79% to 96%, respectively, although the results were not statistically significant. They also found the accuracy of anatomical versus ultrasound guided injection into the submandibular gland to be 50% to 91%, respectively, and the results were statistically significant. This study thus concluded that the ultrasound guided approach was more accurate when compared with the anatomical approach for submandibular gland injections [31]. While in some cases, the ultrasound method was shown to be both safe and effective, there has yet to be a large-scale clinical trial evaluating the superiority of the ultrasound guided approach over the anatomical landmark approach.

## 9. IncobotulinumtoxinA

IncobotulinumtoxinA is the only approved botulinum toxin A for sialorrhea. IncobotulinumtoxinA is also approved for cervical dystonia, upper limb spasticity, and blepharospasm. Inco is manufactured by Merz Pharmaceuticals (Germany) and is available in 50- and 100-unit vials, and it is unique in that it can be stored at room temperature. IncobotulinumtoxinA is the only botulinum product that is stable in the lyophilized form for 4 years at room temperature. Inco differs from the other toxins in that it does not have any complexing proteins and only consists of the active neurotoxin. The risk of spread to unintended muscles and the rate of adverse effects are the same between IncobotulinumtoxinA and the other botulinumtoxinAs. In animal studies, it has been shown that Inco did not lead to the production of serum neutralizing antibodies, likely related to the fact that it does not contain non-toxic accessory proteins(NAPs) [32].

In a pivotal phase 3 trial (SIAXI), Jost et al. conducted the largest prospective, randomized, placebo controlled multicenter study investigating the use of incobotulinumtoxinA for sialorrhea in 184 patients with PD, atypical parkinsonism, CVA, or TBI. Patients were divided into one of three groups: Placebo, 75 U of toxin, and 100 U of toxin. The main endpoint was changes in unstimulated salivary flow rate at 4 weeks. The baseline to 4-week difference in unstimulated salivary flow rate (uSFR) was −0.02 in the 75 U versus placebo and −0.09 in the 100 U versus placebo group. Adverse effects in the treatment group were dry mouth (5.4% vs. 2.7%) and dysphagia (2.7% vs. 0%). The study found sustained efficacy at 16 weeks post-injection. Overall, this pivotal trial resulted in the FDA approving incobotulinumtoxinA 100 U for the treatment of chronic sialorrhea in adult patients [28].

## 10. RimabotulinumtoxinB

RimabotulinumtoxinB is the only approved botulinum toxin B for chronic sialorrhea. RimabotulinumtoxinB is also the only commercially approved botulinum toxin B and is also approved for cervical dystonia. It is manufactured by Myobloc (USA) and is available in 2500, 5000, or 10,000 bottles. The rest of its properties are similar to the other toxins. It has been hypothesized that botulinum toxin B may be more effective at controlling secretory disorders such as sialorrhea. Botulinum toxin B has an increased affinity to cholinergic autonomic neurons, including postganglionic muscarinic receptors such as M_3_ [33]. Moreover, previous studies have shown a relatively high incidence of dry mouth in patients treated with botulinum toxin B for cervical dystonia [33].

In another pivotal phase three trial, Isaacson et al. conducted a randomized trial on the safety and efficacy of RimabotulinumtoxinB for treatment of sialorrhea in adults. In this randomized clinical trial of 187 adults with sialorrhea, RimabotulinumtoxinB injections (2500 U and 3500 U) appeared to statistically significantly reduce sialorrhea versus placebo for patients with Parkinson’s disease, ALS, and CVA. The primary outcome was change in USFR (unstimulated salivary flow rate) and change in clinical global impression of change at four weeks. In this study, both 2500 U and 3500 U resulted in statistically significant results. The treatments with 2500 U and 3500 U had a reduction in USFR from placebo of −0.30 g/min. The mean CGI-C improved in the 2500 U group versus placebo by −1.21, and in the 3500 versus placebo by −1.14; both were statistically significant. Therapeutic benefits were seen as early as 1 week after injection and persisted for 11 to 15 weeks for the 3500 U group. Major adverse effects included dry mouth, dysphagia, and dental caries. This study provided evidence that RimabotulinumtoxinB has efficacy at reducing saliva production and is generally very safe and tolerable [29].

## 11. OnabotulinumtoxinA

OnabotulinumtoxinA is indicated for chronic migraine, upper limb spasticity, cervical dystonia, severe axillary hyperhidrosis, blepharospasm, strabismus, glabellar lines, lateral canthal lines, and overactive bladder. Ona is made by Allergan Inc (USA) and comes in 100 U packaging and must be refrigerated. Its size is about 900 kDa, and constituents include hemagglutinin, human albumin, saccharose, and sodium chloride. Onabotulinumtoxin A induces some degree of antibody induced therapy failure. It has been well studied in cervical dystonia patients, where initial reports of failure were up to 17%, but that estimate has dropped to 1.2% with the more recent batches [34]. Small studies have been conducted that have shown a significant improvement in sialorrhea with minimal side effects, but as of the present, there have been no large studies evaluating the efficacy of OnabotulinumtoxinA on sialorrhea in PD [35,36]. Overall, OnabotulinumtoxinA is the most widely available botulinum toxin and has the largest amount of indications, but currently has no approved indication for sialorrhea. 

## 12. AbobotulinumtoxinA

AbobotulinumtoxinA is indicated for cervical dystonia, glabellar lines, upper limb spasticity in adults, and pediatric limb spasticity. Abobotulinumtoxin A is made by Ipsen (France). It is packaged in 500 U vials, and its constituents are hemagglutinin, human albumin, 20% solution, and lactose. The equivalency ratio between Onabotulinumtoxin A to Abobotulinumtoxin A is about 1:3–4 [27]. There is level A evidence (along with Abobotulinumtoxin A) for treatment of cervical dystonia, and level C for lower limb spasticity. A literature review of 65 patients with 317 sessions compared either injection of Abobotulinumtoxin A 250 U and Rimabotulinumtoxin B 2500 U. They found both agents to be effective at treating sialorrhea and both to have minimal adverse effects. The review concluded both Abobotulinumtoxin A and Rimabotulinumtoxin B were safe, well tolerated, and effective treatments for sialorrhea [37]. There has not been a large randomized control study yet for Abobotulinumtoxin A in the treatment of chronic sialorrhea. 

## 13. Conclusions

Sialorrhea, or drooling, is defined as excessive pooling or spillage of saliva out of the mouth. It is one of the most common complaints affecting up to 80% of Parkinson’s patients [2]; however, it is underrecognized and undertreated. It is also one of the most problematic symptoms related to Parkinson’s disease [3]. Sialorrhea is a prominent symptom in Parkinson’s disease that is both distressing and challenging. It often results in social isolation, embarrassment, depression, skin infections, and aspiration pneumonia. Alternative therapies have proven to be suboptimal, ineffective, or have led to polypharmacy in an at risk population [38]. Two large pivotal stage 3 clinical trials for Incobotulinumtoxin A and Rimabotulinumtoxin B [28,29] showed both the efficacy and safety of both these toxins in the treatment of chronic sialorrhea. Both have now been approved as first line therapy for the treatment of chronic sialorrhea. 

## Figures and Tables

**Table 1:** Comparing the four major botulinum toxins.

Toxins	Brand Name	Indication	Company	Clinical Trial	Dosage	Side-Effect/Cons
**Botulinum toxin A**						
IncobotulinumtoxinA	Xeomin	FDA-approved for chronic Sialorrhea	Merz Pharmaceuticals (Germany)	SIAXI	100 U	Dry mouth, dysphagia [28]
OnabotulinumtoxinA	Botox	No FDA approval for Sialorrhea	Allergan US		-	Unknown
AbobotulinumtoxinA	Dysport	No FDA approval for Sialorrhea	Ipsen (France)		-	Unknown
**Botulinum toxin B**						
RimabotulinumtoxinB	Myobloc	FDA-approved for chronic Sialorrhea	Myobloc (USA)	Isaacson et al.	2500 U and 3500 U	Dry mouth, dysphagia, and dental caries [29]
